# A Low-Cost Sensor Network for Real-Time Thermal Stress Monitoring and Communication in Occupational Contexts

**DOI:** 10.3390/s22051828

**Published:** 2022-02-25

**Authors:** Markus Sulzer, Andreas Christen, Andreas Matzarakis

**Affiliations:** 1 Chair of Environmental Meteorology, Department of Earth and Environmental Sciences, Faculty of Environment and Natural Resources, University of Freiburg, D-79085 Freiburg, Germany; andreas.christen@meteo.uni-freiburg.de (A.C.); andreas.matzarakis@dwd.de (A.M.); 2 Research Centre Human Biometeorology, German Meteorological Service, Stefan-Meier-Str. 4, D-79104 Freiburg, Germany

**Keywords:** environmental monitoring system, human thermal comfort, low-cost sensors, physiologically equivalent temperature, sensor network

## Abstract

The MoBiMet (Mobile Biometeorology System) is a low-cost device for thermal comfort monitoring, designed for long-term deployment in indoor or semi-outdoor occupational contexts. It measures air temperature, humidity, globe temperature, brightness temperature, light intensity, and wind, and is capable of calculating thermal indices (e.g., physiologically equivalent temperature (PET)) on site. It visualizes its data on an integrated display and sends them continuously to a server, where web-based visualizations are available in real-time. Data from many MoBiMets deployed in real occupational settings were used to demonstrate their suitability for large-scale and continued monitoring of thermal comfort in various contexts (industrial, commercial, offices, agricultural). This article describes the design and the performance of the MoBiMet. Alternative methods to determine mean radiant temperature (*T_mrt_*) using a light intensity sensor and a contactless infrared thermopile were tested next to a custom-made black globe thermometer. Performance was assessed by comparing the MoBiMet to an independent mid-cost thermal comfort sensor. It was demonstrated that networked MoBiMets can detect differences of thermal comfort at different workplaces within the same building, and between workplaces in different companies in the same city. The MoBiMets can capture spatial and temporal differences of thermal comfort over the diurnal cycle, as demonstrated in offices with different stories and with different solar irradiances in a single high-rise building. The strongest sustained heat stress was recorded at industrial workplaces with heavy machinery.

## 1. Introduction

The thermal and radiative environment has a strong impact on human thermal comfort, performance, and health [1]. In particular, during heatwaves, the thermal environment can have a negative effect on the economy [2] by reducing work capacity [3] and productivity [4], and disrupting production processes [5]. Heat stress can cause heatstroke, heat exhaustion, and multiple related medical implications [6]. Although deaths and several illnesses can be caused directly by heat stress in extreme conditions; in most situations they are related to the worsening of pre-existing health conditions [6]. Indirect psychological and social burdens including stress, anxiety, and depression can also be health consequences [7]. Heat also leads to a reduction in the ability to work and an increase in injury-related mortality and morbidity [8]. Heat stress appears at outdoor and indoor workplaces and can be caused by natural or artificial factors, or a combination of both [3]. Globally, heatwaves have become more frequent and intense nearly everywhere on earth since the 1950s [9]. In recent years, the frequency of record-breaking heat waves in Europe has also increased [10]. Any further rise in global warming will lead to a noticeable increase in the frequency and intensity of hot extremes and heatwaves [9]. In the near future, heatwaves in Europe will be longer, more intense, and occur more frequently [11]. For example, the European heat waves of 2003 and 2015 strongly contributed to the top-two records of summer mortality in southwest Germany (Baden-Württemberg) in the period from 1968 to 2015 [10]. In the next decades, 2015-like heat waves are expected to take place nearly every second summer in southwest Germany, according to the projections of regional climate models [10]. This causes new challenges for the health, safety, and wellbeing of workers [8,12]. Raising awareness of heat stress can be a simple way to promote behavioral changes in those affected [7].

Air temperature (*T_a_*) alone is not the sole variable that affects human thermal comfort [13,14]. In addition, the human energy balance is affected by vapor pressure (*ρ_v_*), mean radiant temperature (*T_mrt_*), wind velocity (*v*) [13,14], and personal variables of clothing and activity [14,15]. Multiple thermal indices are used in human biometeorological studies that take the four meteorological variables into account, while the personal variables are kept fixed [16,17]. Appropriate thermal indices are based on the exchange of energy between body surfaces and the internal heat production of humans [14]. The physiologically equivalent temperature (PET) is one of the most widely used thermal indices based on the human energy balance, and can be applied for outdoor and indoor locations [17,18,19]. The unit of PET is °C, which makes the index easy to understand for everyone, including those who are not familiar with human-biometeorological terminology [14,20], for example, employees or decision makers [21].

To raise awareness of potentially challenging thermal conditions and to assist the development of useful mitigation and adaptation strategies, local and room-specific surveys on thermal comfort must be used [22]. This information can be provided by portable systems deployed for short investigation periods at locations of concern [22]. Statistical information on the frequency of thermal comfort levels at different workplaces could also help to maintain occupational health standards and form room-specific adaptation measures. Possible adaptation measures to reduce heat stress at workplaces can be artificial air cooling by the installation of additional air-conditioning devices, exterior solar shading, shifting the time of day for working, wearing ventilated clothes, taking more breaks, increasing forced ventilation, or changing the industrial structure, for example, by the mechanization of human work [8,23,24]. However, there are no affordable systems available to continuously monitor different workplaces in companies and communicate heat stress to employees.

In recent years, low-cost sensors have become increasingly popular in environmental sciences [25]. The increased usage of low-cost sensors is driven, among other factors, by the reduced cost of microcontrollers and environmental sensors as well as by the open science movement [25]. Compared to state-of-the-art commercial sensors, low-cost sensors have poorer robustness and accuracy, and calibrations are often necessary, but because of the lower costs, more sensors can be used and much finer spatial coverage is possible [25]. Several low-cost sensor systems have already been proposed and used for the determination of thermal comfort indoors or outdoors [26,27,28,29,30].

Vargas et al. [26] built a prototype of a low-cost sensor to measure thermal comfort outdoors at a fixed place. The device was powered by a battery and a solar panel [26]. The system was used to measure meteorological variables needed for the calculation of human thermal comfort over five months at two urban locations in Valencia (Spain) [31]. Chiesa et al. [27] built a prototype of a low-cost device to measure the thermal comfort of pedestrians at fixed or movable monitoring points, powered by a battery. The mobile measurements of the system were compared to a professional thermal comfort station and to a survey with 20 participants and ten monitoring points at three times on one day around downtown Turin (Italy) [27]. Kimmling and Hoffmann [29] developed the “Comfort Monitoring Station” (CoMoS) to determine indoor human thermal comfort. A field test was performed with ten CoMoS devices to measure thermal comfort at different positions inside a sloping lecture hall with 160 seats during one afternoon at a conference in Luxembourg [29]. The “nano Environmental Monitoring System” (nEMoS) was developed by Salamone et al. [28], and is a low-cost sensor system to measure indoor thermal comfort and air quality [28,32]. As part of an experimental study, eight nEMoS devices measured the thermal comfort of workers on the top of their desks in five offices and two floors for a four-week period inside a single building near Milan (Italy) [33]. In addition to the environmental measurements by nEMoS, biometric variables measured by wearable devices and feedback from the workers via a web-based survey were used to develop an individual thermal comfort control strategy [33]. nEMoS sent data to a cloud server via Wi-Fi [28]. Mthunzi et al. [30] made a prototype of an ultra-low-cost thermal comfort monitoring station for indoor use and compared the data measured by their monitoring station in a climate chamber to measurements by conventional thermal comfort sensors.

All five mentioned low-cost thermal comfort monitoring systems determine *T_mrt_*, *T_a_*, and *ρ_v_* [26,27,28,29,30]. All systems use a custom-made black globe thermometer to calculate *T_mrt_*. Four of the systems also measure *v*, with three of the systems using a hot-wire anemometer and one using a cup anemometer [26,27,28,29,30].

Building on the above studies, the goal of the current study was to develop a larger network of 100+ low-cost devices to determine human thermal comfort simultaneously in different occupational contexts at various indoor and semi-outdoor locations continuously, for a time period of over one year. The collected data will not only benefit occupational health assessments, but also provide valuable datasets for evaluating operational heat-health warning systems (e.g., German Meteorological Service). The following design principles were imperative. The data should be readily communicated and accessible for employees and decision makers, and therefore, the real-time and statistical data on human thermal comfort must be determined and visualized on the device, as well as on a website. The device should be small, cost-effective, easy to install, and not be disruptive to the work flow. Given the size and challenge of positioning a black globe thermometer, alternative calculation options for *T_mrt_* should also be considered.

The specific aims of this article are to:

1. Document the low-cost sensors used, their integration into a human thermal comfort monitoring system, and communication;
2. Assess the uncertainty in measuring different environmental variables used for thermal comfort calculations and the uncertainty in the integral thermal comfort relative to commercial state-of-the art sensors;
3. Assess the difference in calculating *T_mrt_* using different combinations of sensors—with and without a black globe thermometer;
4. Demonstrate whether the network of devices can resolve differences between workplaces in the same building and between workplaces in different companies in the same city.

## 2. Materials and Methods

### 2.1. Sensor Design

The proposed low-cost sensor for thermal stress monitoring in occupational contexts is named the “Mobile Biometeorological System” (MoBiMet), because it is small and easily deployable in large numbers. The MoBiMet is based on a single-board computer Raspberry Pi Zero WH (Raspberry Pi (Trading) Limited, Cambridge, UK). Connected to the single-board computer are sensors for the measurement of *T_a_*, *ρ_v_*, *T_mrt_*, *v* (only at semi-outdoor locations), thermal incident radiation (*LW*), and light level (*L*), mounted in a 3D-printed enclosure. The MoBiMet is powered by a 5 V adapter via a micro-USB cable. It can also be powered by a commercial battery pack for a few days. Because the MoBiMets are measuring thermal comfort at workplaces where power is readily available and for a long time period, we used power supplies in this study. During the design process, different low-cost sensors were tested for accuracy (details can be found in the Appendix A, Appendix B, Appendix C, Appendix D and Appendix E). State-of-the-art research grade sensors were used as references.

#### 2.1.1. Air Temperature and Humidity

A combined low-cost capacitive humidity and thermistor sensor was used for *T_a_* and humidity measurements on the MoBiMet (DHT22, Aosong Electronics Co., Ltd., Guangzhou, China [34]). The DHT22 was selected out of six different low-cost *T_a_* and humidity sensors by comparing them in a climate chamber and in an outdoor Stevenson Screen (Appendix A). All DHT22 sensors were calibrated in real-word conditions and in a climate chamber (WEISS BA SB22-300, Weiss Klimatechnik GmbH, Reiskirchen, Germany) for a *ρ_v_* range of 8.3–24.3 hPa and a *T_a_* range between −10–50 °C. The DHT22 has been used in multiple low-cost projects as thermometer and hygrometer [27,28].

#### 2.1.2. Mean Radiant Temperature

For the determination of *T_mrt_*, a custom-made black globe thermometer was built. The black globe thermometer is made of a thermometer (DS18B20, Maxim Integrated Products, Inc., San Jose, CA, USA) in a hollow stainless-steel ball with a thickness of 0.0004 m and a diameter of 0.05 m, painted with matte black acrylic paint. Different globes were tested for the design of the black globe thermometer next to a commercial black globe (Appendix B), similar to the study from Vargas-Salgado et al. [35]. DS18B20 thermometers have been used in similar projects inside of black globe thermometers [26,29,35,36] and have been calibrated in the same climate chamber for the temperature range −5–50 °C. *T_mrt_* was calculated from measured globe temperature (*T_g_*), *T_a_*, and *v*, according to Equation (A1) (see Appendix B).

For an alternative way to determine *T_mrt_* (see Section 2.2), the MoBiMet is also equipped with a light intensity sensor (BH175, ROHM Semiconductor GmbH, Willich, Germany) and a contactless infrared thermopile (IR) sensor (MLX90615, Waveshare Electronics, Shenzhen, China). Three different sensors were tested for the IR sensor (Appendix C). The light intensity sensor was compared against an ISO 9060 spectrally flat class A pyranometer (CMP21, Kipp & Zonen, Delft, The Netherlands).

#### 2.1.3. Wind Velocity

At indoor workplaces, measurement of *v* is generally so low that it is not considered in the determination of human thermal comfort [37]. The mean values of *v* are below 0.1 m s^−1^ at most indoor locations [38]. In indoor environments, in contrast to *v,* the natural convection at the clothed surface of the human body is more relevant [39]. At semi-outdoor locations, a cup anemometer (Windsensor WS, Eltako GmbH, Fellbach, Germany) was attached to the MoBiMet. The Windsensor WS was calibrated against a state-of-the-art propeller anemometer (05130, R. M. Young Company, Traverse City, MI, USA, see Appendix D). The starting speed of the Windsensor WS was approximately 0.5 m s^−1^, so for most indoor workplaces, the Windsensor WS was not used. For the calculation of PET and *T_mrt_* at indoor locations without a Windsensor WS attached, *v* is set to 0.1 m s^−1^ [40]. At semi-outdoor locations, *v* was also set to a minimum of 0.1 m s^−1^ if *v*, measured by the Windsensor WS, was 0.0 m s^−1^.

#### 2.1.4. Enclosure and Sensor Screens

3D-printed enclosures are often used for low-cost systems in environmental meteorology [26,41]. Different designs of the enclosure and sensor screens for the MoBiMet were tested. The design criteria were to achieve a compact enclosure, yet provide enough ventilation and heat dissipation to minimize the impact of the waste heat of the Raspberry Pi on the measurements. Furthermore, the radiation error when the sensor is in the sun should be as low as possible. Appendix E provides a detailed description of the different designs of the enclosure and the sensor screens tested, and their performance for accurate *T_a_* measurements.

**Figure 1:**
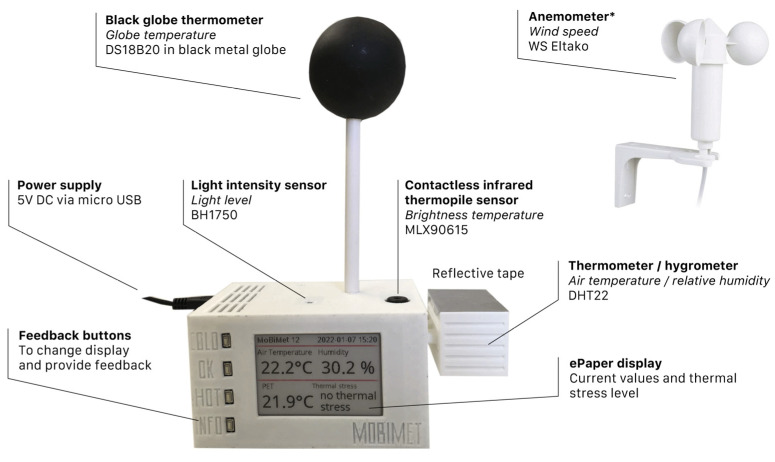
Final design of the MoBiMet (* only at semi-outdoor locations).

In Figure 1, the final implemented design of the MoBiMet enclosure is shown. The thermal incident radiation and light intensity sensors point upwards. The black globe thermometer is mounted on a 0.09 m plastic (Acrylnitril-Styrol-Acrylester, ASA) tube screwed into the housing at the top. A MoBiMet has dimensions 0.15 m × 0.08 m × 0.2 m, including the black globe thermometer, and weighs approximately 175 g. The screen of the *T_a_* and humidity sensor has inclined slats to enable ventilation of the DHT22, as well as reflective tape to mitigate the influence of direct solar radiation on the sensor. The slats are 0.002 m wide and have a slope of 33.5° through the 0.002 m thick screen wall. There are additional ventilation holes on the underside of the screen. For further details, the 3D model can be found in the Supplementary Materials. The DHT22 is as far away from the Raspberry Pi as possible. Without the anemometer, the material for one MoBiMet costs approximately €75 (including the anemometer, approximately €125).

#### 2.1.5. Display and Communication

An ePaper display (2.7inch e-Paper HAT, Waveshare Electronics, Shenzhen, China), is connected to the Raspberry Pi to continuously visualize the measured data on the device and provide an immediate assessment of thermal comfort. Each sensor is sampled once every five min, stored locally on a SD card, and sent to a MYSQL database on a server over Wi-Fi or LoRaWAN (Long Range Wide Area Network). LoRaWAN is used for data transfer in companies without Wi-Fi, such as in large industrial facilities. For data transfer with LoRaWAN, the IoT LoRa Node pHAT for Raspberry Pi (Pi Supply (Nebra Ltd.), Bells Yew Green, UK) was added to the MoBiMet for an additional €28. 

The Raspberry Pi calculates PET (°C) on the device based on the Python script by Walther and Goestchel [42]. The meteorological input variables for the PET calculations are *T_a_*, *ρ_v_*, *T_mrt_*, and *v*. By default, PET is calculated by the MoBiMet for a standardized person (male, 35 years, 1.75 m, 75 kg) [43] with reference clothing of 0.9 clo and a work metabolism of 80 W (the two thermophysiological factors correspond to light activity in a business suit [14,19,44]). The personal data, however, can be changed on the device. The values of PET are then classified into nine thermophysiological stress levels (Table 1) [20]. The current thermophysiological stress level and the latest values of *T_a_* (°C), relative humidity (*RH*) (%), and PET are printed on the display. The language of the display can be set to English, French, or German. All Python scripts running on the MoBiMet can be found in the Supplementary Materials.

**Figure 2:**
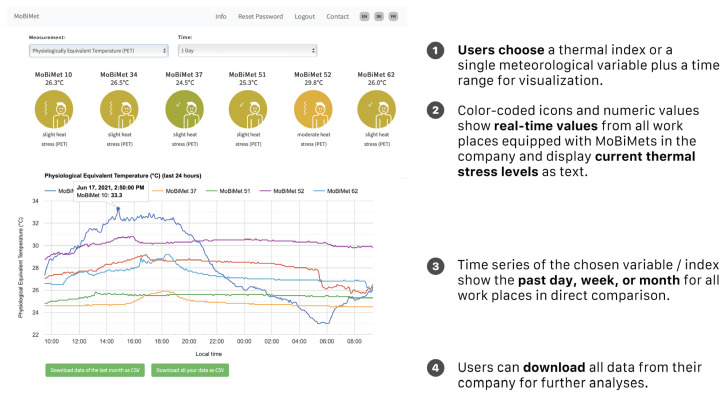
Layout of the MoBiMet website.

Users can access, graph, and compare current and all historical data of one or multiple MoBiMets in their company on a secure and private MoBiMet website (Figure 2). To access to the data of the MoBiMets within a company, users have logins with individual usernames and passwords. The users can choose which meteorological variable and time period they want to visualize on the graph. The page also shows the current values of a chosen variable and the current thermophysiological stress levels of each MoBiMet in a company. The data can also be downloaded directly at the website as a CSV file for further use.

**Figure 3:**
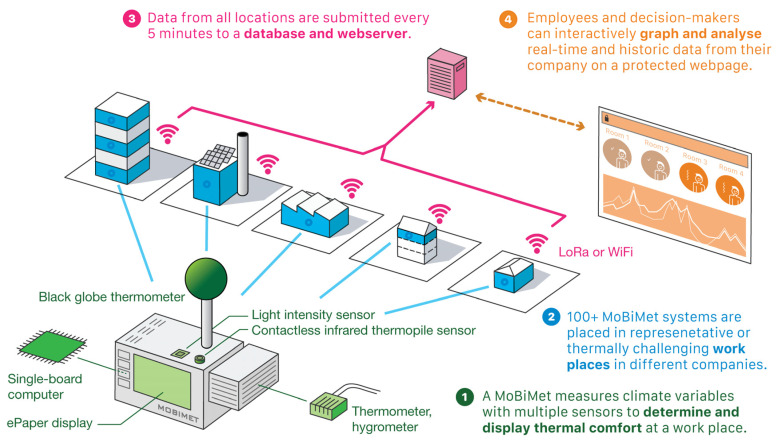
Structure of the MoBiMet network (at semi-outdoor locations, an additional cup anemometer is attached to the MoBiMet).

In total, 120 MoBiMets were built and installed at different workplaces in 35 companies in Switzerland, France, and Germany (Figure 3). The number of MoBiMets in a single company ranged from one to eleven. MoBiMets in offices were consistently placed on top of a desk at a height of approximately 0.7 m. At other workplaces that are not offices, MoBiMets were installed at the working place where they do not disturb the workers, near a power supply on the wall or on top of structures. When possible, the MoBiMets were installed at a typical working height of 1.5 m; when the MoBiMet would be disruptive at 1.5 m, it was installed higher. The maximum height of an installed MoBiMet was 2.5 m.

### 2.2. Different Methods to Determine T_mrt_

Custom-made black globe thermometers are often used for the determination of *T_mrt_* [26,32,45]. However, black globe thermometers have the disadvantage of being sensitive and require significant additional space (Figure 1). In this project, in addition to a black globe thermometer, alternative approaches to infer indoor *T_mrt_* were tested using a light intensity sensor and an IR sensor. The IR sensor gives the value of the incident *LW* (W m^−2^), which is translated to a brightness temperature (*T_IRT_)* according to the Stefan–Boltzmann law, and the light intensity sensor is used as a surrogate for solar radiation (*SW*). The measured *L* (lx) of the light intensity sensor was fitted with a linear regression with an upper bound (due to its saturation effect) to the measurement of global radiation of a CMP21 Pyranometer to approximate *SW* (W m^−2^) at the top the MoBiMet (1):

(1) SW ≈0.03887 L

When *L* is higher than 15,000 lx, *SW* is set to 600 W m^−^^2^. Because the MoBiMets IR and *L* measurements are only performed in one direction—up—the six-directional method for mean radiant flux density (*S_str_*) used in previous human biometeorology studies [46,47,48] was modified to calculate an approximation of *T_mrt_* (°C) (2):

(2) $$T_{mrt}=\sqrt[{4}]{\frac{S_{str}}{\left(\varepsilon_{p\;}\sigma \right)}}-273.15\;\;\;\;\;\;\;\;\;\;\;\;\;\;\;\;\;\;\;\;\;\;\;\;\;\;\;\;\;\;\;\;\;\;S_{str}\approx F\;\alpha_{k}\;SW+\varepsilon_{p}\;LW$$

It is assumed that in indoor environments, *LW* is isotropic from all six directions and *SW* comes primarily from above. Two values of the angular weighting factor *F* are tested for the *S_str_* calculation. *F* = 0.167 represents one direction of a sphere and *F* = 0.06 for the radiation from above, for a standing person [47]. σ is the Stefan–Boltzmann constant (5.67·10^−8^ W m^−2^ K^−4^), $\alpha_{k}$ is the absorption coefficient for short-wave radiation (standard value 0.7), and $\varepsilon_{p}$ is the emissivity of the human body (standard value 0.97) [47].

*T_mrt_* calculated from the custom-made black globe thermometer and the two versions of *T_mrt_* calculated by the IR and *L* were compared to *T_mrt_* measured by a Kestrel 5400 Heat Stress Tracker (Nielsen-Kellerman Co., Boothwyn, PA, USA), which uses a commercial black globe thermometer to determine *T_mrt_* and which was placed next to a MoBiMet in an office in Freiburg (Germany) for two weeks in August 2021. The mobile Kestrel 5400 Heat Stress Tracker can be used to determine human thermal comfort [49,50] and costs about seven times as much as the material for the MoBiMet (without anemometer). Furthermore, *T_IRT_* inferred directly from the IR sensor and *T_a_* were set as *T_mrt_*, resulting in a total of five different estimates of *T_mrt_* by the MoBiMet (Table 2), which were then compared to *T_mrt_* provided by the Kestrel. The black globe thermometer of the Kestrel 5400 Heat Stress Tracker has a diameter of 0.025 m [51]. Measured *T_g_* is converted internally by the Kestrel 5400 Heat Stress Tracker to the equivalent value measured inside a standard globe [51]. A standard globe has a diameter of 0.15 m, is painted black, has an emissivity $\varepsilon_{g}$ of 0.95, and is made of thin-walled (0.0004 m) copper [51,52].

### 2.3. Evaluation of the Complete Sytem

Between 4 August and 17 August 2021, a Kestrel 5400 Heat Stress Tracker was installed next to a MoBiMet in an office during a typical summer situation to evaluate the MoBiMet system under realistic conditions. The measurements of the Kestrel were recorded every ten min, with *T_a_* ranging between 20.1 °C and 29.0 °C and *RH* from 36.7–67.6% recorded by the Kestrel in the observed time period.

### 2.4. Comparison of the Thermal Comfort Levels between Different Workplaces

To check if multiple MoBiMets can detect differences in thermal comfort between different offices inside a single building and between workplaces of different companies in a city, the individual readings and the calculated thermal stress determined by MoBiMets were compared between different rooms in multiple floors and different exposures in an office building in Freiburg, and between workplaces at different companies in Freiburg during sunny days between 2 September and 9 September 2021. To illustrate the influence of outdoor weather conditions, PET values in the office building were also analyzed on cloudy days between 28 August and 31 August 2021. 

## 3. Results and Discussion

### 3.1. Performance of the Different T_mrt_ Estimation Methods

**Figure 4:**
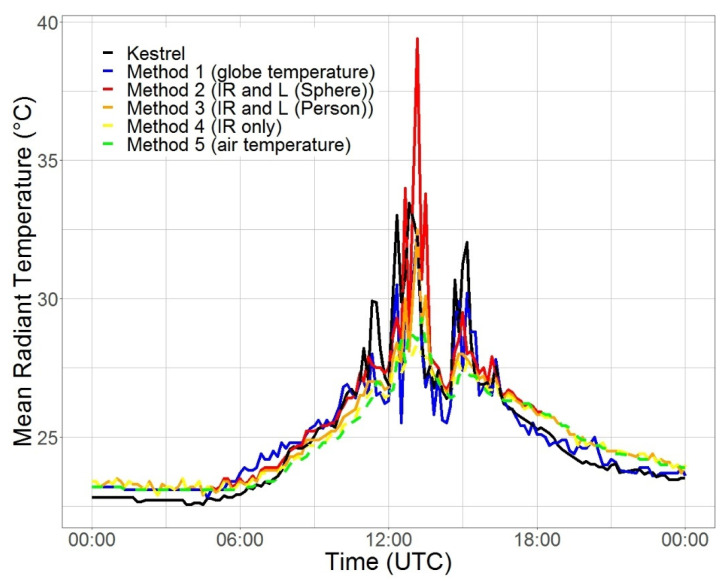
Mean radiant temperature (°C) in an office in Freiburg on 8 August 2021, calculated by a Kestrel 5400 Heat Stress Tracker and the five different calculations methods (Table 2) using data from the MoBiMet.

In Figure 4, the daily cycle of *T_mrt_* measured by the Kestrel and estimated by five different methods with the MoBiMet is shown for 8 August 2021. At night, *T_mrt_* calculated by Methods 2, 3, and 4, using IR and *L* or just IR, showed the same values and were nearly the same as Method 5, because there is no shortwave radiation and *T_IRT_* measured by the IR sensor is close to *T_a_,* whereas *T_mrt_* usually is slightly lower than *T_a_* during the night [52]. Compared to the Kestrel, the values of *T_mrt_* were slightly overestimated during the night. The calculated *T_mrt_* values of Method 1 using the custom-made black globe thermometer were closest to the values of the Kestrel at night. During the day, with direct sunlight on both systems and hence the workplace, the different methods to calculate *T_mrt_* show larger differences. Most of the methods to calculate *T_mrt_* from the data of the MoBiMet underestimate *T_mrt_* when direct solar radiation hits the sensors during the day.

**Figure 5:**
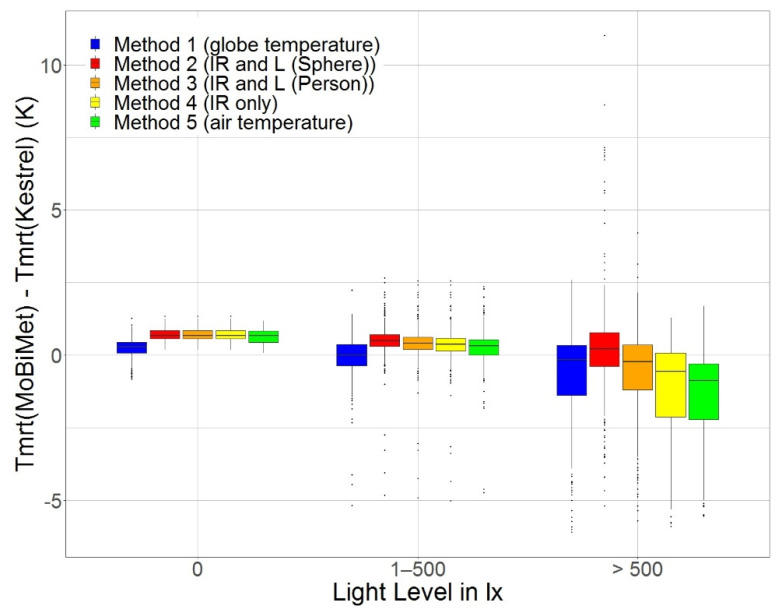
Boxplots of the difference in mean radiant temperature (*T_mrt_*) (K) between the reported measurements of a Kestrel 5400 Heat Stress Tracker and the MoBiMet using the five different calculation methods (Table 2). Sorted by the light level (lx) measured by the MoBiMet in an office in Freiburg between 4 August and 17 August 2021.

Figure 5 shows boxplots of the differences between *T_mrt_* according to the Kestrel and the different calculation methods of the MoBiMet, sorted by *L* for the values 0 lx (660 measurements), 1–500 lx (863 measurements), and >500 lx (231 measurements).

When the values of *L* are 0 lx and 1–500 lx, *T_mrt_* determined by the MoBiMet with Method 1 using the custom-made black globe thermometer shows the smallest differences from the *T_mrt_* values of the Kestrel. At *L* > 500 lx, Method 1 shows the second smallest differences after the *T_mrt_* values calculated by Method 3 using IR and *L* (Person). With increasing *L*, the range of *T_mrt_* differences of all methods compared to the Kestrel also increases. A possible explanation for the underestimation of *T_mrt_* by Method 1 compared to the Kestrel are the different diameters of the globes. Smaller globes have a faster response time, but the effect of *T_a_* and *v* on *T_g_* is also larger due to convective heat exchange, which reduces the accuracy of *T_mrt_* [53]. A source of error in the calculation of *T_mrt_* of the Kestrel and Method 1 of the MoBiMet is the assumption that *v* indoors is set to a constant value of 0.1 m s^−1^ in Equation (A1) (Appendix B). Another possible source of error is the inaccurate conversion of *T_g_* to the equivalent of the standard globe internally performed by the Kestrel. This conversion works best at *v* > 1 m s^−1^ [51], which is outside the range of the conditions found during the comparison. Overall, the values of *T_mrt_* determined with Method 1 using the custom-made black globe thermometer showed the smallest values for the root mean square error (RMSE), mean bias error (MBE), mean absolute error (MAE), and mean squared error (MSE) (Table 3).

The RMSE of all methods compared to the Kestrel are between 0.85 K and 1.03 K. The low errors of Method 5, which took *T_a_* as *T_mrt,_* confirm that indoors, most of the time, *T_mrt_* and *T_a_* are close [39,54], but when direct solar radiation hits the sensors, the differences are substantial [48]. A dataset from 1 August to 30 September 2021 of all MoBiMets deployed in different contexts shows that the mean absolute difference of *T_mrt_* calculated with the globe and with both Methods 2 and Method 3 using the IR and *L* is 0.96 K. For further study, *T_mrt_* determined with Method 1 employing the custom-made black globe thermometer was used for the PET calculation. A limitation of Methods 2 and 3 is the usage of a single IR and *L* sensor. The methods could be improved by using multiple IR and *L* sensors pointing in different directions.

### 3.2. Evaluation of the System for Thermal Comfort Modelling

**Figure 6:**
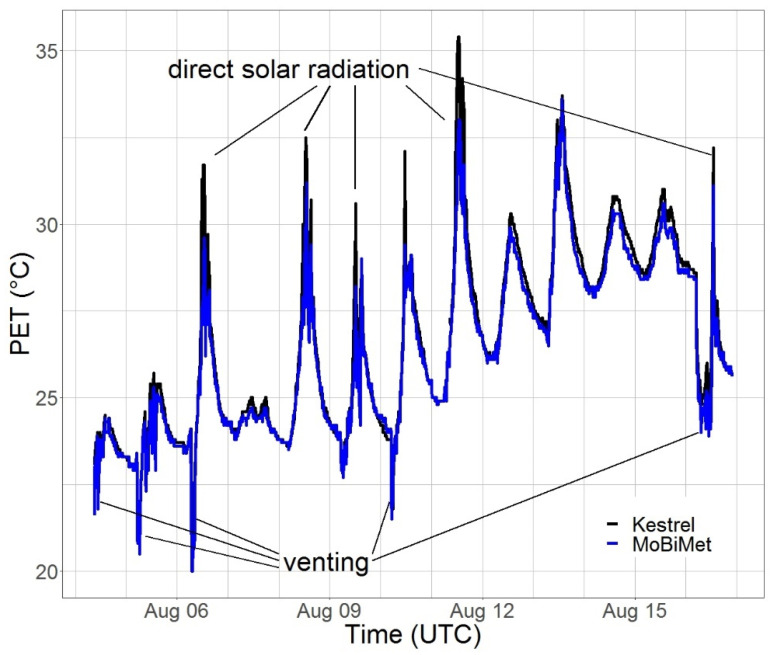
Physiologically equivalent temperature (PET) (°C) measured by a Kestrel 5400 Heat Stress Tracker (black) and a MoBiMet (blue) in an office in the city center of Freiburg between 4 August and 17 August 2021.

Illustrative PET values determined by the Kestrel 5400 Heat Stress Tracker and the MoBiMet over a period of two weeks in an office in Freiburg are shown in Figure 6.

The RMSE, MBE, MAE, and MSE of the different meteorological variables and the PET of the MoBiMet relative to the Kestrel are listed in Table 4.

Overall, with an RMSE of 0.57 K between the PET values of the MoBiMet and the Kestrel for the two-week period, it can be concluded that the data of the two systems fit together well. When the systems are exposed to direct solar radiation, the MoBiMet underestimates *T_a_*, *T_mrt_*_,_ and PET values compared to the Kestrel. This can be partly explained by the radiation error of *T_a_* measurements of the Kestrel 5400 Heat Stress Tracker, which has an exposed, unshielded thermistor [49,50], leading to higher *T_a_* and PET values of the Kestrel during direct solar radiation. On the other hand, the radiation shield of the MoBiMet can lead to a small delay when *T_a_* changes quickly. Another explanation for the underestimation of PET during the influence of direct solar radiation is the underestimation of *T_mrt_* by the MoBiMet (Section 3.1). A source of error may be the assumption that *v* is only 0.1 m s^−1^. The RMSE of *T_a_* is reduced by 0.377 K and of *ρ_v_* by 0.09 hPa by the calibration. This underlines the importance of sensor-individual calibrations when using low-cost sensors, as confirmed in other studies [25].

### 3.3. Resolving Different Thermal Comfort Levels between Different Workplaces

**Figure 7:**
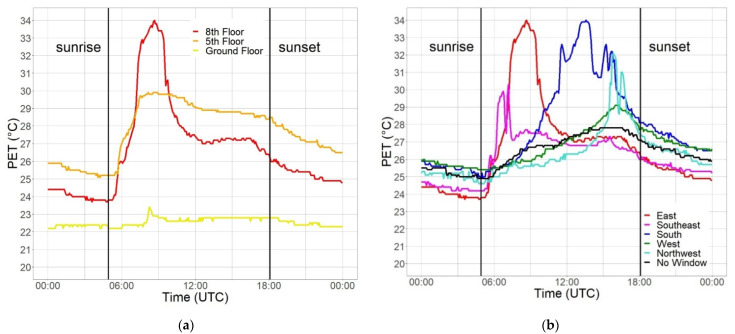
Physiologically equivalent temperature (PET) (°C) measured by MoBiMets in the same office building in the city center of Freiburg on 5 September 2021: (**a**) all sensors in offices with windows exposed to the east on different floors; (**b**) on the same floor (eighth) with different window exposures.

Figure 7 shows the measured diurnal cycles of PET on a sunny day in the beginning of September 2021 from multiple MoBiMets in an office building in the inner city of Freiburg. The different systems show large differences between MoBiMets located on different floors and in offices with different exposures.

Figure 7a shows that after sunrise, PET values increase in all offices exposed to the east (on the ground floor, fifth floor, and eighth floor), and they peak mid-morning at the same time, as all three MoBiMets were placed directly behind an east-facing window. However, the amplitude of the increase in PET is largest on the eighth floor and decreases with height in the building. The PET values on 5 September 2021 on the ground floor show the smallest diurnal amplitude, ranging only between 22.2 °C and 23.4 °C, because the ground floor room’s window is shaded by trees and other buildings. During the same day, the PET values on the fifth floor range from 25.1–29.9 °C, and on the eighth, from 23.7–34.0 °C. The constantly higher PET values in the afternoon and in the night in the office on the fifth floor compared to the eighth and ground floor is explained by the poor ventilation of the room [55]. The windows and door in the office on the fifth floor are permanently closed.

Figure 7b shows PET values in five offices on the eighth floor with different exposures, and the hallway in between. The workplaces show large differences in the diurnal cycle because of the different orientations of the windows and, consequently, the timing and intensity of solar radiation [56]. The peak values of PET in the offices exposed to the east and southeast occur in the morning, in the office in the south at noon, and in the offices exposed to west, northwest, and in the hallway, in the afternoon. The PET values of the hallway (no windows) have the smallest diurnal amplitude of all sensors placed on the eighth floor, with values between 24.9 °C and 27.8 °C, as there is no influence of direct solar radiation. The PET values in the office exposed to the southeast increase at the same time as the one exposed to the east, shortly before 06:00 (UTC), but the values are only between 24.2 °C and 30.3 °C. The smaller amplitude of the PET values in the office exposed to the southeast compared to the office exposed to the east can be explained by the position of the MoBiMets inside the offices [57]. In the office exposed to the east, the MoBiMet was placed directly behind the window and is strongly influenced by direct solar irradiance, whereas the MoBiMet in the office exposed to the southeast was placed in the middle of the room. The closer the position is to a window, the more frequently heat stress occurs [58]. After 14:00 (UTC), the smallest PET values can be found in the offices exposed to the east and northeast. These offices no longer receive direct solar irradiation. The PET values of the office exposed to the west rise less than the PET values of the office exposed to northwest in the afternoon, but the PET values also descend less in the evening and at night. The PET values in the office exposed to the west range from 25.4–29.1 °C, whereas the PET values in the office exposed to the northwest are between 24.6 °C and 32.1 °C. This can be explained by a smaller window in the office exposed to the west [59]. The MoBiMet in the office exposed to the northwest is shaded by a computer screen at approximately 16:00 (UTC), which explains the sharp local dip of the PET values during the late afternoon.

**Figure 8:**
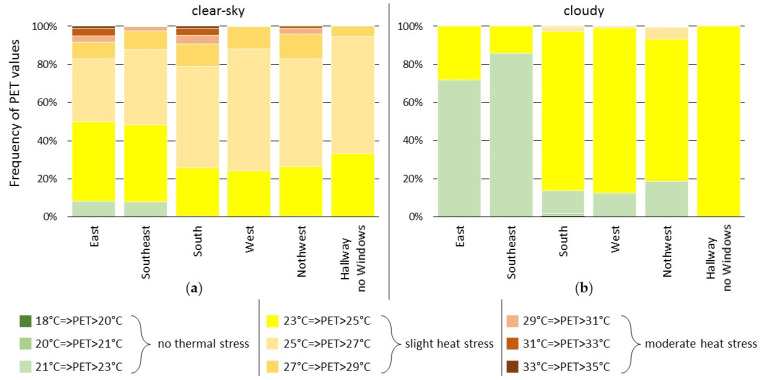
Distribution of physiologically equivalent temperature (PET) values measured by MoBiMets in the same office building in the city center of Freiburg with different exposures on the eighth floor: (**a**) during a clear-sky week between 2 September and 9 September 2021; (**b**) during cloudy days between 28 August and 31 August 2021.

Figure 8 contrasts the thermal comfort during a week with warm and sunny weather from 2 September to 9 September 2021, and a period with predominantly cloudy weather, between 28 August and 31 August 2021. The distribution of PET values measured by the same six MoBiMets on the eighth floor of the office building are shown. During cloudy weather, constant slight heat stress was observed in the hallway. In contrast, during the sunny week, the hallway is the only location on the eighth floor where no moderate heat stress occurs. The most frequent comfortable thermal conditions on the eighth floor can be found in the office exposed to the southeast during cloudy days. No thermal stress occurred in this office 86% of the 24 h time (and 75% of the daytime working hours between 08:00 (UTC) and 18:00 (UTC) local time) during the cloudy days. During sunny days, the highest PET values were determined by MoBiMets exposed to direct solar radiation, because the human thermal comfort indoors is highly impacted by direct solar radiation through a window [60,61]. It can be confirmed that window exposure to the south causes the worst thermal conditions [58], because moderate heat stress is most likely to occur in the office exposed to the south by a margin of 10%.

**Figure 9:**
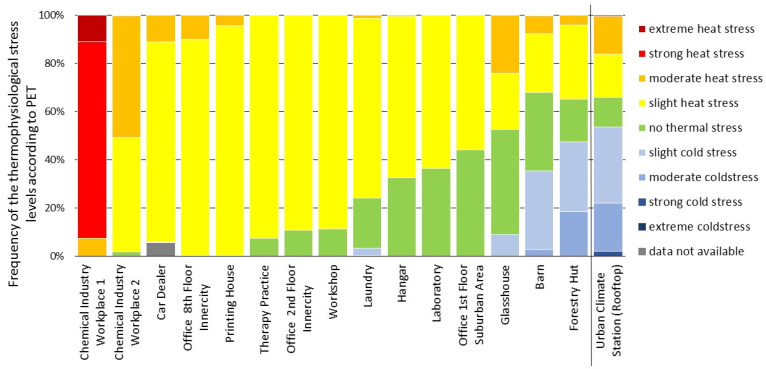
Distribution of thermal comfort according to physiologically equivalent temperature (PET) measured by MoBiMets at different workplaces in Freiburg and at the urban climate station during a clear-sky week between 2 September and 9 September 2021.

Figure 9 shows the frequency distribution of thermophysiological stress levels according to PET determined by MoBiMets at workplaces in different companies and buildings in the city of Freiburg, Germany, during the same sunny time period from 2 September to 9 September 2021.

Compared to the subtle variations of heat stress frequencies in the office building (Figure 8), there are more significant differences found in different companies and contexts (Figure 9). Most prominently, Workplaces 1 and 2, located in a chemical industry plant are near industrial machinery that produces a significant amount of waste heat. At Workplace 1, the PET values range between 31.9 °C and 43.2 °C. It was the only workplace where extreme heat stress occurred in this week. Strong heat stress was recorded 82% of the time. At Workplace 2, the impact of the waste heat is limited, but still, 50% of the observed time moderate heat stress occurred, and the frequency of comfortable thermal conditions was only 2%.

At a car dealership and in a printing house, heat stress occurred continuously during the entire observed time period between 2 September and 9 September 2021. The workplace with the most comfortable thermal conditions was an office on the first floor in a suburban area of Freiburg. The PET values ranged from 19.4–27.5 °C. For 44% of the observed time period, the thermal conditions were comfortable and 56% of the time, only slight heat stress occurred. In an office on the second floor in the inner city of Freiburg, less comfortable conditions (11%) and more slight heat stress (89%) appeared, and the PET values ranged from 20.2–27.1 °C. The MoBiMets in the two offices were not affected by direct solar irradiation. A possible explanation for the higher PET values in the city center is the influence of the urban heat island and reduced venting in dense urban areas [62].

Except for the laundry, cold stress only occurred at workplaces outdoors or semi-outdoors, due to nocturnal cooling and increased ventilation.

To put the observed indoor and semi-outdoor heat stress into context, PET was also calculated for data from a conventional urban climate station located outdoors on a rooftop of a high-rise building at a height of 51 m above ground in Freiburg (48°00′04″ N; 7°50′55″ E). At the urban climate station, cold stress was most frequent by 54%, comfortable thermal conditions only occurred 12% of the observed time period during the sunny days, and heat stress was detected 34% of the time during the same period. The urban climate station is the only site where strong cold stress occurred during the considered time period. The wide range of PET values at the urban climate station can be explained by the influence of direct solar radiation due to a large sky view factor (for the high PET values [63,64]), and the higher values of *v* due the lack of obstacles and the height of the roof top (low PET values [65]). At all workplaces except the barn and the forestry hut, heat stress was more frequent than at the urban climate station. The effects of heat on the health of humans depend on the duration, frequency, and intensity with which they are exposed to heat stress [7]. The differences between the frequency of the thermal stress levels according to PET at the urban climate station and the MoBiMet measurements at the different workplaces show the need for more detailed information about thermal comfort at indoor locations, because frequency and intensity of thermal stress indoors cannot be adequately determined by conventional measurements outdoors.

## 4. Conclusions

The current contribution documented a prototype low-cost sensor network made up of devices called “MoBiMets”, each measuring *T_a_*, *ρ_v_*, *T_g_*, *T_IRT_*, and *L*. The MoBiMets only measure *v* at semi-outdoor locations. MoBiMets calculate thermal comfort, communicate it in real-time on their ePaper display, and transmit data to a sever, where it can be graphed on a webpage. Information about the thermal comfort at workplaces is important, because it can be linked to occupational health and safety [14]. The MoBiMets demonstrate the potential for low-cost sensors for determining and continuously communicating human thermal comfort in occupational contexts. Their performance, considering the small size of the system and the low cost of the material in comparison with mid-cost sensors such as Kestrel 5400 Heat Stress Trackers, is acceptable. The long-term stability of the measurements has yet to be determined. More expensive sensors, a bigger enclosure, or a black globe thermometer with a larger diameter could improve the accuracy of the human thermal comfort determined by the MoBiMet. The MoBiMets could also be used in clinics, schools, residential contexts, or other places in non-occupational contexts with vulnerable populations for short- or long-term monitoring of thermal comfort. The MoBiMet network can help to identify heat stress, which can be the first step in reducing heat vulnerability [7]. It can raise awareness of heat stress, which can lead to changes in the behavior of those affected [7], e.g., changing to a less-stressful work place or shifting working time, if possible.

We have shown that MoBiMets can reveal significant differences in the magnitude and timing of thermal stress in a single building, within one company or between multiple ones. Because *T_mrt_* has the highest impact on human thermal comfort [40,66], whether the MoBiMet is exposed to direct solar radiation or not is very important. *T_mrt,_* as well as *v*, have large spatial variabilities [14]. Therefore, the exact position of each MoBiMet has to be adjusted so that it most properly reflects the position of the worker. In any analysis and comparison, when contrasting multiple sensors, details on solar radiation are key. A light sensor helps to determine when the sensor/workplace is exposed to direct sunlight.

*T_mrt_* is the most complex variable impacting human thermal comfort [14,48]. Integral radiation measurements are best suited for the determination of *T_mrt_* [45,47], and this would be a better reference for the selection of the custom-made black globe thermometer and the comparison between the different alternative determination methods of *T_mrt_*. Alternative methods to approximate *T_mrt_* with IR and light intensity sensors could be further improved by IR and/or *L* measurements in multiple directions with multiple sensors. Other *L* sensors could also be tested to improve the alternate methods of determining *T_mrt_*. By using one of the alternate methods of determining *T_mrt_*, the size of the MoBiMet could be significantly reduced, but in its current state, the *T_mrt_* calculated by the measurements of the custom-made black globe thermometer shows the lowest errors.

Unfortunately, in the current version, *v* is not recorded at many indoor locations because of the cost of the *v* sensor and the fact that the selected cup anemometer is not responsive to typical *v* at indoor locations [17]. A cup anemometer is also not appropriate for low *v* semi-outdoor environments. In future versions, the MoBiMet could be expanded to include a more sensitive hot wire or ultrasonic anemometer for *v* measurements, especially for indoor locations, as have been used in similar projects [27,28,29,32]. This could improve the calculation of *T_mrt_* using the black globe thermometer and the calculation of PET, notably when airing a room or during *v* below the threshold of the cup anemometer at semi-outdoor locations. Another interesting addition would be a low-cost air quality or CO_2_ sensor to inform workers about the need to vent indoor air [67,68]. Because the enclosure of the MoBiMet is not waterproof, determination of human thermal comfort is only possible at indoor and semi-outdoor workplaces. As a future improvement, a waterproof enclosure could enable the usage of the MoBiMet at outdoor locations, as demonstrated with similar low-cost systems [26,31]. Another improvement would be to design a more power efficient version of the MoBiMet using a low-power microcontroller instead of the Raspberry Pi to enable long-term observations using a battery. In addition, a module could be added with which data can be transmitted via the cellular network.

In future steps, the MoBiMet network will be integrated into heat-health warning systems to minimize the negative health impacts of heat episodes [69]. Heat-health warning systems are based on weather observations and forecasts, and MoBiMets will help to translate outdoor observations and forecasts to occupational indoor environments [70,71]. A large number of MoBiMets, for example, could be used to model or parameterize typical time lags and dampening functions between outdoor and indoor temperatures [71], or to evaluate simple physical schemes that predict indoor thermal comfort.

## Figures and Tables

**Table 1:** Physiologically equivalent temperature (PET) range for the different levels of thermophysiological stress on standardized human beings for defined activity and clothing [20].

PET	Thermophysiological Stress Level
<4 °C	extreme cold stress
4–8 °C	strong cold stress
8–13 °C	moderate cold stress
13–18 °C	slight cold stress
18–23 °C	no thermal stress
23–29 °C	slight heat stress
29–35 °C	moderate heat stress
35–41 °C	strong heat stress
>41 °C	extreme heat stress

**Table 2:** Different calculation methods for mean radiant temperature (*T_mrt_*) tested on the MoBiMet devices.

#	Method	Calculation	Sensors Used
1	Globe temperature	Equation (A1) (Appendix B)	Using black globe thermometer, air temperature thermistor, and cup anemometer (at semi-outdoor locations)
2	IR and *L* (Sphere)	Equation (2) with *F* = 0.167	Using IR and light intensity sensor
3	IR and *L* (Person)	Equation (2) with *F* = 0.06	Using IR and light intensity sensor
4	IR only	*T_mrt_* = *T_IRT_*	Using IR sensor
5	Air temperature	*T_mrt_* = *T_a_*	Using air temperature thermistor

**Table 3:** Root mean square error (RMSE), mean bias error (MBE), mean absolute error (MAE), and mean squared error (MSE) for the different methods of the mean radiant temperature (*T_mrt_*) (K) from a MoBiMet in an office, relative to an adjacent Kestrel 5400 Heat Stress Tracker.

#	Method	RMSE	MBE	MAE	MSE
1	Globe temperature	0.850	−0.031	0.517	0.722
2	IR and *L* (Sphere)	1.030	0.543	0.761	1.060
3	IR and *L* (Person)	0.888	0.371	0.658	0.788
4	IR only	0.986	0.273	0.681	0.973
5	Air temperature	0.973	0.178	0.664	0.946

**Table 4:** Root mean square error (RMSE), mean bias error (MBE), mean absolute error (MAE), and mean squared error (MSE) for air temperature (*T_a_*) (K), mean radiant temperature (*T_mrt_*) (K), vapor pressure ($\rho_{v}$) (hPa), and physiologically equivalent temperature (PET) (K) from a MoBiMet in an office relative to an adjacent Kestrel 5400 Heat Stress Tracker.

Variable	RMSE	MBE	MAE	MSE
*T_a_* (K)	0.296	−0.127	0.193	0.088
*ρ_v_* (hPa)	0.291	−0.121	0.194	0.085
*T_mrt_* (K)	0.849	−0.027	0.517	0.721
PET (K)	0.570	−0.306	0.342	0.325

**Figure A1:**
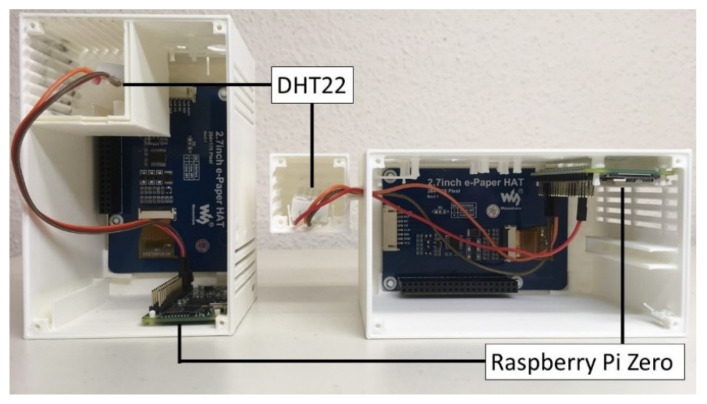
Vertical (**left**) and horizontal (**right**) version of the enclosure.

## Data Availability

The data presented in the results of this study are openly available at https://doi.org/10.5281/zenodo.5846454 (accessed on 20 February 2022).

## Supplementary Materials

The Python scripts and the 3D model of the enclosure can be found at https://github.com/markus1203/MoBiMet/tree/master/Python_Scripts_and_3D_Model (accessed on 20 February 2022).

## Appendix A. Performance of Different Low-Cost Air Temperature/Humidity Sensors

The following air temperature (*T_a_*) and relative humidity (*RH*) low-cost sensors were considered for the MoBiMet system: AM2320 (Aosong Electronics Co., Ltd., Guangzhou, China), DHT22 (Aosong Electronics Co., Ltd., Guangzhou, China), MCP9808 (Adafruit Industries, New York, NY, USA), BME680 (Bosch Sensortec GmbH, Reutlingen, Germany), BME280 (Bosch Sensortec GmbH, Reutlingen, Germany). Two sensors of each model were tested except for the AM2320, where only a single sensor was tested. The MCP9808 measures *T_a_* only. BME680 and BME280 are also able to measure barometric pressure, which is not used in the current application.

For the evaluation of *T_a_* measurements, all sensors were run next to a Fluke Calibration Thermometer 1502 (Fluke Corporation, Everett, WA, USA) in a climatic chamber WEISS BA SB22-300 (Weiss Klimatechnik GmbH, Reiskirchen, Germany) for a *T_a_* range from −10–40 °C. The climate cabinet was set to hold every 5 °C step between this range for 3 h. The tested sensors measured *T_a_* each minute and the reference sensor each second.

For the evaluation and calibration of the humidity sensors, all sensors were tested inside a Stevenson Screen located outdoors next to a Campbell Scientific CS215A Temperature and Humidity probe (Campbell Scientific Inc., Logan, UT, USA) for one week. In order to obtain a measure for humidity that does not depend on *T_a_*, vapor pressure (*ρ_v_*) (hPa) was calculated from *T_a_* and *RH* via the saturated vapor pressure (*ρ_v,s_*) (hPa), using the Clausius–Clapeyron equation [72].

For both *T_a_* and *ρ_v_* measurements, a linear regression was performed on a randomly selected half of the values against the reference sensors to obtain sensor-specific calibration coefficients. The other half of the values of the tested sensors were calibrated with the calibration coefficients. The root mean square error (RMSE), mean bias error (MBE), and mean absolute error (MAE) were determined for the raw (unchanged) and calibrated data of the tested sensors in comparison to the values of the reference sensors.

All tested sensors fulfil the expectations for *T_a_* measurements. RMSE, MBE, and MAE are smaller than 0.1 K for all calibrated sensors (Table A1). After calibration, the two DHT22 sensors showed the smallest errors for *ρ_v_* (Table A2). Hence, the DHT22 was used as the *T_a_* and humidity sensor for the MoBiMet. It was the most accurate for humidity and also performed well for *T_a_*. It was the second cheapest tested sensor, and worked reliably during the tests. Unfortunately, after a few months of operation outdoors, DHT22 humidity measurements seem to drift very rapidly to high values of humidity, some of them staying at a *RH* of 100%. At indoor workplaces, measurements of six MoBiMets were checked after six months against a Campbell Scientific CS215A Temperature and Humidity probe and were still reliable. The mean RMSE of the six MoBiMets for *RH* is 0.7%.

**Table A1:** Individual calibration coefficients (offset and slope), root mean square error (RMSE), mean bias error (MBE), and mean absolute error (MAE) for calibrated and raw values of all tested air temperature sensors.

Sensor	Calibration Coefficients		RMSE (Calibrated) (K)	RMSE (Raw) (K)	MBE (Calibrated) (K)	MBE (Raw) (K)	MAE (Calibrated) (K)	MAE (Raw) (K)
	Offset	Slope						
AM2320	−0.131	1.021	0.063	0.458	−0.003	−0.266	0.051	0.391
DHT22 (#1)	−0.086	1.025	0.068	0.593	0.004	−0.388	0.052	0.501
DHT22 (#2)	−0.177	1.025	0.072	0.542	−0.001	−0.303	0.056	0.465
HTU21D (#1)	0.427	1.001	0.063	0.459	−0.001	−0.454	0.045	0.454
HTU21D (#2)	0.670	1.002	0.061	0.662	−0.001	−0.659	0.044	0.659
MCP9808 (#1)	0.055	1.003	0.058	0.144	0.000	−0.119	0.045	0.128
MCP9808 (#2)	0.224	1.002	0.064	0.263	−0.001	−0.254	0.049	0.254
BME680 (#1)	0.360	0.988	0.056	0.265	0.001	−0.117	0.041	0.208
BME680 (#2)	0.925	0.981	0.070	0.669	0.001	−0.562	0.051	0.562
BME280 (#1)	1.325	0.990	0.068	1.162	0.000	−1.146	0.060	1.146
BME280 (#2)	1.142	0.989	0.071	0.960	−0.001	−0.936	0.063	0.936

**Table A2:** Individual calibration coefficients (offset and slope), root mean square error (RMSE), mean bias error (MBE), and mean absolute error (MAE) for the calibrated and raw values of the vapor pressure of the tested air humidity sensors.

Sensor	Calibration Coefficients		RMSE (Calibrated) (hPa)	RMSE (Raw) (hPa)	MBE (Calibrated) (hPa)	MBE (Raw) (hPa)	MAE (Calibrated) (hPa)	MAE (Raw) (hPa)
	Offset	Slope						
AM2320	0.595	0.782	0.476	2.247	0.005	2.162	0.405	2.162
DHT22 (#1)	0.075	1.003	0.102	0.146	−0.002	−0.103	0.074	0.125
DHT22 (#2)	0.036	0.968	0.107	0.330	−0.001	0.308	0.075	0.309
HTU21D (#1)	0.388	0.967	0.131	0.144	0.003	−0.039	0.088	0.099
HTU21D (#2)	0.321	0.982	0.132	0.191	0.003	−0.137	0.090	0.161
BME680 (#1)	−0.379	1.044	0.204	0.227	−0.002	−0.079	0.157	0.189
BME680 (#2)	−0.367	1.055	0.200	0.290	−0.001	−0.196	0.153	0.253
BME280 (#1)	0.986	1.017	0.139	1.150	−0.002	−1.141	0.102	1.41
BME280 (#2)	0.860	0.998	0.132	0.855	−0.001	0.845	0.097	0.845

## Appendix B. Performance of Different Black Globe Thermometer Variants

To measure mean radiant temperature (*T_mrt_*), a number of custom-made black globe thermometers were built and tested. In all cases, the temperature inside the globes was measured by a DS18B20 (Maxim Integrated Products, Inc., San Jose, CA, USA), which is referred to as globe temperature (*T_g_*). The DS18B20 sensors were calibrated in the climate chamber according to the same procedure as outlined for the DHT22 sensors in Appendix A. For the globes, different materials, sizes, and paints were tested. Hollow stainless-steel balls with a thickness of 0.0004 m and hollow plastic balls made of polypropylene with a thickness of approximately 0.001 m and with diameter of 0.025 m and 0.05 m each were painted with matte black acrylic paint and matte black acrylic spray paint. A commercial black table tennis ball made of polypropylene with a diameter of 0.04 m was also tested with matte black acrylic paint, matte black acrylic spray paint, and without paint (Table A3).

**Table A3:** Different globes tested for the determination of globe temperature (*T_g_*).

Number	Material	Diameter (m)	Paint
1	Stainless steel	0.05	Matte black acrylic spray paint
2	Plastic	0.05	Matte black acrylic spray paint
3	Plastic	0.05	Matte black acrylic spray paint
4	Plastic	0.025	Matte black acrylic spray paint
5	Plastic	0.025	Matte black acrylic spray paint
6	Plastic	0.05	Matte black acrylic paint
7	Stainless steel	0.025	Matte black acrylic spray paint
8	Stainless steel	0.05	Matte black acrylic paint
9	Table tennis ball	0.04	Matte black acrylic paint
10	Plastic	0.025	Matte black acrylic paint
11	Table tennis ball	0.04	None
12	Table tennis ball	0.04	Matte black acrylic paint
13	Plastic	0.05	Matte black acrylic paint
14	Table tennis ball	0.04	Matte black acrylic spray paint
15	Table tennis ball	0.04	None
16	Table tennis ball	0.04	Matte black acrylic spray paint
17	Stainless steel	0.025	Matte black acrylic paint

The globes were tested on the roof-top of a high-rise building next to a Testo black copper globe (Testo SE & Co. KGaA, Titisee-Neustadt, Germany) with a diameter of 0.15 m, and a Kestrel 5400 Heat Stress Tracker (Nielsen-Kellerman Co., Boothwyn, PA, USA), on 18 November 2020, a sunny day with low wind velocity (*v*). Inside the Testo black copper globe was also a DS18B20 temperature module. The mean value of *v* measured on 18 November 2020 was 0.5 m s^−1^ and the maximum was 3.5 m s^−1^, *T_a_* ranged between 5 °C and 16 °C, and the peak of the global radiation was recorded as 402 W m^−2^. All measurements were collected every minute and five-min mean values were calculated for further comparison. *T_mrt_* was calculated with Equation (A1) [47]:

(A1) $$T_{mrt}=\sqrt[{4}]{{\left(T_{g}+273.15\right)}^{4}+\frac{1.1\times 10^{8}v^{0.6}}{\varepsilon_{g}D^{0.4}}\left(T_{g}-T_{a}\right)}-273.15$$

The parameter $1.1\times 10^{8}$ and the wind exponent $v^{0.6}$ together are the convection coefficient. *T_a_* (°C) was derived by a CS215A temperature and humidity probe (Campbell Scientific Inc., Logan, UT, USA) of a co-located climate station, and *v* (m s^−1^) was measured by the co-located Kestrel 5400 Heat Stress Tracker. The convection coefficient was adapted to the different globes [47]. *D* is the diameter (m) and $\varepsilon_{g}$ is the globe emissivity. $\varepsilon_{g}$ was set to 0.95 [48].

The values of $T_{g}$ of the Testo copper globe were used to determine the reference for *T_mrt_*. For a randomly selected half of the data points (12 h), the convection coefficient was optimized. The other half of the values (12 h) was used to test the optimized formula. RMSE, MBE, and MAE were determined for the data of *T_mrt_* calculated with Formula (A1) (raw). *T_mrt_* calculated with the adapted convection coefficients (optimized) of the tested sensors is shown in comparison to the values of the reference sensors in Table A4.

**Table A4:** Adapted convection coefficient (where *v* is the wind velocity (m s^−1^)) and root mean square error (RMSE), mean bias error (MBE), and mean absolute error (MAE) for the optimized and raw values of the tested mean radiant temperature sensors (K).

Number	Optimized Convection Coefficient	RMSE (Optimized)	RMSE (Raw)	MBE (Optimized)	MBE (Raw)	MAE (Optimized)	MAE (Raw)
1	$1.51\times 10^{8}v^{0.40}$	3.104	5.441	−1.381	−2.400	1.713	3.391
2	$1.75\times 10^{8}v^{0.37}$	3.257	6.724	−1.486	−2.773	1.831	4.453
3	$1.68\times 10^{8}v^{0.37}$	3.143	6.419	−1.473	−2.729	1.815	4.168
4	$1.72\times 10^{8}v^{0.34}$	3.257	7.089	−1.551	−3.006	1.961	4.651
5	$1.76\times 10^{8}v^{0.37}$	3.007	6.930	−1.168	−2.678	1.746	4.739
6	$1.71\times 10^{8}v^{0.38}$	2.571	6.224	−1.120	−2.482	1.484	4.205
7	$1.67\times 10^{8}v^{0.38}$	2.696	6.399	−1.106	−2.548	1.589	4.325
8	$1.54\times 10^{8}v^{0.46}$	1.956	4.610	−0.130	−1.332	0.954	3.392
9	$1.70\times 10^{8}v^{0.32}$	2.789	6.823	−2.052	−3.307	2.301	4.270
10	$1.57\times 10^{8}v^{0.26}$	3.658	7.273	−2.912	−4.052	3.103	4.568
11	$1.77\times 10^{8}v^{0.26}$	3.813	7.897	−3.055	−4.093	3.242	4.856
12	$1.79\times 10^{8}v^{0.32}$	2.502	7.022	−1.732	−3.138	2.042	4.534
13	$1.58\times 10^{8}v^{0.34}$	1.910	5.656	−2.635	−1.293	1.580	3.603
14	$1.73\times 10^{8}v^{0.32}$	2.281	6.667	−2.946	−1.477	1.867	4.326
15	$1.91\times 10^{8}v^{0.29}$	3.080	7.785	−3.517	−2.149	2.564	5.005
16	$1.77\times 10^{8}v^{0.33}$	2.258	6.756	−2.893	−1.377	1.841	4.457
17	$1.65\times 10^{8}v^{0.31}$	2.276	6.648	−2.898	−1.249	1.838	4.391

Number 8, the globe of stainless steel with a diameter of 0.05 m and painted with matte black acrylic paint, performed best, and hence was used for the MoBiMets. The empirical adapted convection coefficient in Formula (A1) is $1.54\cdot 10^{8}v^{0.46}$. Number 8 is the only globe with a MAE under 1 K when using the optimized convection coefficient.

## Appendix C. Performance of Low-Cost Infrared Thermopile Sensors

Three contactless infrared thermopile (IR) sensors were considered for the design of the MoBiMet. The evaluated IR sensors are the MLX90615 (Waveshare Electronics, Shenzhen, China), the TMP007 (Adafruit Industries, New York, NY, USA), and the TMP006 (Adafruit Industries, New York, NY, USA). Two replications of each infrared sensor were tested.

The sensors were pointed at a container containing an ice-water mixture for 30 min, which was then exchanged with 55 °C warm water gradually cooling down over 30 min. A Fluke Calibration Thermometer 1502 (Fluke Corporation, Everett, WA, USA) was inserted in the water bath to compare the water temperature with the measurements of the contactless IR sensors. The brightness temperatures of the IR sensors were compared to the Fluke Calibration Thermometer 1502.

The two MLX90615 showed the smallest errors, and the MLX90615 was used for all MoBiMets (Table A5).

**Table A5:** Root mean square error (RMSE), mean bias error (MBE), and mean absolute error (MAE) for the values of the tested infrared temperature sensors.

Sensor	RMSE (K)	MBE (K)	MAE (K)
TMP007 (#1)	5.059	−0.310	4.536
TMP007 (#2)	6.367	−0.675	5.988
TMP006 (#1)	5.614	−1.225	5.098
TMP006 (#2)	5.088	−1.185	4.631
MLX90615 (#1)	1.330	−0.520	0.844
MLX90615 (#2)	1.391	−0.703	0.944

## Appendix D. Calibration of the Cup Anemometer

The low-cost wind velocity (*v*) sensor Windsensor WS (Eltako GmbH, Fellbach, Germany) was tested at the Hartheim Forest Research Site [73,74] of the University of Freiburg next to a wind monitor 05130 (R. M. Young Company, Traverse City, MI, USA) at 30 m above ground level. The signal was sampled each 60 s over one month. A linear regression with *v* of the reference sensor and the count of the signal of the Windsensor WS provided generic calibration coefficients for all Windsensor WS. Only values where the signal of the Windsensor WS was at least 1 count were used for the calibration. *v* can be calculated with the count of the Windsensor WS and the calibration coefficients.

The starting speed of the Windsensor WS is approximately 0.5 m s^−1^. The *v* (m s^−1^) can be calculated with the following Formula (A2).

(A2) v=0.005361·n+0.485451 

where n is the count of the wind signal of the Windsensor WS over 60 s. The residual RMSE is 0.399 m s^−1^, the MBE −0.004 m s^−1^, and the MAE 0.262 m s^−1^.

## Appendix E. Performance of Different Enclosures and Screens

The enclosure for the MoBiMet system is made of white polylactide (PLA) or white acrylonitrile butadiene styrene (ABS) constructed with a 3D printer. The different materials have no impact on the measurements.

Different designs of the enclosure were tested. The design criteria were to achieve a compact enclosure, yet provide enough space for the display, allow for ventilation and heat dissipation, and minimize the impact of the significant waste heat of the Raspberry Pi Zero WH (Raspberry Pi (Trading) Limited, Cambridge, UK) on the measurements (runs on average at ~45 °C and can heat up to 70 °C). Further, the radiation error when the sensor is in the sun should be as low as possible.

The different designs of the enclosure were tested with measurements of *T_a_* of calibrated DHT22 sensors mounted in the enclosure inside a Stevenson Screen next to a Campbell Scientific CS215A Temperature and Humidity probe for 40 h and outside, next to the Stevenson Screen, for seven hours on a sunny day.

Two general alignments of the enclosure design were tested—vertical and horizontal (Figure A1)—where the Raspberry Pi is placed in the top right corner in the horizontal version and in the bottom right corner in the vertical version. The DHT22 sensors were placed in an extra box or a plastic tube on the left of the enclosure in the vertical version and inside the enclosure or on top of the enclosure in the vertical version of the enclosure (Table A6). The extra boxes have inclined slats and on some of the boxes reflective tape was glued to prevent the DHT22 from receiving direct solar radiation while enabling ventilation. In one version, additionally, a small fan was tested for forced ventilation and a sensor without any housing was used as a reference.

**Table A6:** Different tested versions of the enclosure.

Version	Alignment	Position of the Air Temperature Sensor	Ventilation Slats	Reflective Tape	Fan
1	Vertical	In extra box inside	Top and right	X	
2	Vertical	In extra box inside	Top and right		
3	Vertical	In extra box inside	Top and right		X
4	Vertical	In extra box on top	Front, left and right		
5	Horizontal	In extra box on the right	Left, right, front, back, bottom, top		
6	Vertical	In plastic tube (Ø = 25 mm) on top	Front and back	X	
7	Vertical	In plastic tube (Ø = 20 mm) on top	Front and back	X	
8	Vertical	In plastic tube (Ø = 40 mm) on top	Front and back	X	
9	Without enclosure				
10	Horizontal	In an extra box on the right	Left, right, front, back, bottom	X	
11	Horizontal	In an extra box on the right	Left, right, front, back, bottom, top		
12	Horizontal	In plastic tube (Ø = 25 mm) on the right	Front and back	X	
13	Horizontal	In plastic tube (Ø = 20 mm) on the right	Front and back	X	

Most of the vertical enclosures performed poorer than the horizontal enclosures because the waste heat of the Raspberry Pi ascended and affected the sensor. Enclosure Version 10 showed the smallest errors, as it contained the temperature and humidity measurements inside an extra box on the right of the main enclosure (Table A7). The RMSE, MBE, and MAE are all under 0.1 K. Version 10 was therefore chosen for the MoBiMet. When exposed to direct solar radiation, the measurements of all versions of the enclosure overestimate *T_a_*. The enclosure with the fan provided the smallest errors under direct sunlight, but greater errors than most of the other versions of the enclosure inside the Stevenson Screen. Another reason that the version with the fan was not used for the MoBiMet, was that the fan started to make noises after a few days.

**Table A7:** Root mean square error (RMSE), mean bias error (MBE), and mean absolute error (MAE) for the calibrated values of the air temperature (K) in the enclosures in a Stevenson Screen (inside) and next to a Stevenson Screen (outside) exposed to direct sunlight.

Version	RMSE (Inside)	MBE (Inside)	MAE (Inside)	RMSE (Outside)	MBE (Outside)	MAE (Outside)
1	1.042	0.913	0.913	6.803	4.524	4.533
2	1.448	1.418	1.418	4.370	3.335	3.335
3	0.689	0.657	0.657	1.617	0.876	1.083
4	0.428	0.300	0.341	4.801	2.865	3.099
5	0.233	0.195	0.214	2.886	1.742	1.925
6	0.593	0.500	0.501	2.420	1.710	1.776
7	1.241	1.180	1.183	2.908	1.830	1.994
8	0.407	0.372	0.375	2.608	1.867	1.887
9	0.231	0.192	0.203	4.781	2.848	3.058
10	0.099	−0.022	0.075	2.245	1.650	1.673
11	0.181	−0.125	0.138	3.185	1.860	2.074
12	0.374	−0.329	0.329	1.805	1.215	1.300
13	0.299	−0.253	0.253	2.728	1.456	1.513
